# High-Throughput Metabolomics Applications in Pathogenesis and Diagnosis of Valvular Heart Disease

**DOI:** 10.31083/j.rcm2406169

**Published:** 2023-06-08

**Authors:** Daniel W. Mutithu, Jennifer A. Kirwan, Henry A. Adeola, Olukayode O. Aremu, Evelyn N. Lumngwena, Lubbe Wiesner, Sebastian Skatulla, Richard Naidoo, Ntobeko A. B. Ntusi

**Affiliations:** ^1^Division of Cardiology, Department of Medicine, Faculty of Health Sciences, University of Cape Town and Groote Schuur Hospital, 7925 Cape Town, South Africa; ^2^Cape Heart Institute, Faculty of Health Sciences, University of Cape Town, 7925 Cape Town, South Africa; ^3^Extramural Unit on Intersection of Noncommunicable Diseases and Infectious Diseases, South African Medical Research Council, 7501 Cape Town, South Africa; ^4^Metabolomics Platform, Berlin Institute of Health at Charité-Universitätsmedizin Berlin, 10117 Berlin, Germany; ^5^Max-Delbrück-Center (MDC) for Molecular Medicine in the Helmholtz Association, 13125 Berlin, Germany; ^6^Hair and Skin Research Laboratory, Division of Dermatology, Department of Medicine, University of Cape Town, 7925 Cape Town, South Africa; ^7^Institute of Infectious Diseases and Molecular Medicine (IIDM), University of Cape Town, 7925 Cape Town, South Africa; ^8^Division of Clinical Pharmacology, Department of Medicine, University of Cape Town, 7925 Cape Town, South Africa; ^9^Computational Continuum Mechanics Research Group, Department of Civil Engineering, Faculty of Engineering and the Built Environment, University of Cape Town, 7925 Cape Town, South Africa; ^10^Division of Anatomical Pathology, Department of Pathology, University of Cape Town, and National Health Laboratory Services, 7925 Cape Town, South Africa; ^11^Cape Universities Body Imaging Centre, Faculty of Health Sciences, University of Cape Town, 7925 Cape Town, South Africa; ^12^Wellcome Centre for Infectious Disease Research, Faculty of Health Sciences, University of Cape Town, 7925 Cape Town, South Africa

**Keywords:** metabolomics, valvular heart disease, rheumatic valve disease, degenerative valve disease, mass spectrometry

## Abstract

High-throughput metabolomics techniques are a useful tool to understand many disease conditions including cardiovascular disease such as valvular heart disease(s) (VHD). VHD involves damage to heart valves, mostly presenting as stenosis, regurgitation or prolapse and can be classified into degenerative, rheumatic, congenital, or prosthetic valve disease. Gaps remain in our understanding of the pathogenesis of the common VHD. It is now fitting to place into perspective the contribution of metabolomics in the mechanism of development, diagnosis, and prognosis of VHD. A structured search for metabolomics studies centred on human VHD was undertaken. Biomarkers associated with the pathogenesis of bicuspid aortic valve disease, mitral valve disease, rheumatic heart disease, and degenerative aortic valve stenosis are reviewed and discussed. In addition, metabolic biomarkers reported to prognosticate patient outcomes of post-valve repair or replacement are highlighted. Finally, we also review the pitfalls and limitations to consider when designing metabolomics studies, especially from a clinician’s viewpoint. In the future, reliable and simple metabolic biomarker(s) may supplement the existing diagnostic tools in the early diagnosis of VHD.

## 1. Introduction

Metabolomics is the high-throughput comprehensive measurement and investigation of small molecules (substrates, intermediate metabolites, and products) within a biosystem [1]. Metabolomics has become an important technique in clinical research for biomarker discovery for several disease conditions and phenotypes, e.g., heart failure, cancer and chronic kidney disease and contributes towards personalised medicine [1, 2, 3, 4, 5, 6]. Metabolic phenotyping has led to the idea of a “metabotype”, i.e., a group of individuals with similar metabolic profiles which can be used in precision screening, diagnosis, and prognosis [7]. In addition to disease phenotypes causing perturbations of the metabolome, gut or oral microbiome dysbioses have also been associated with changes in the metabolome [8].

**Fig. 1.**
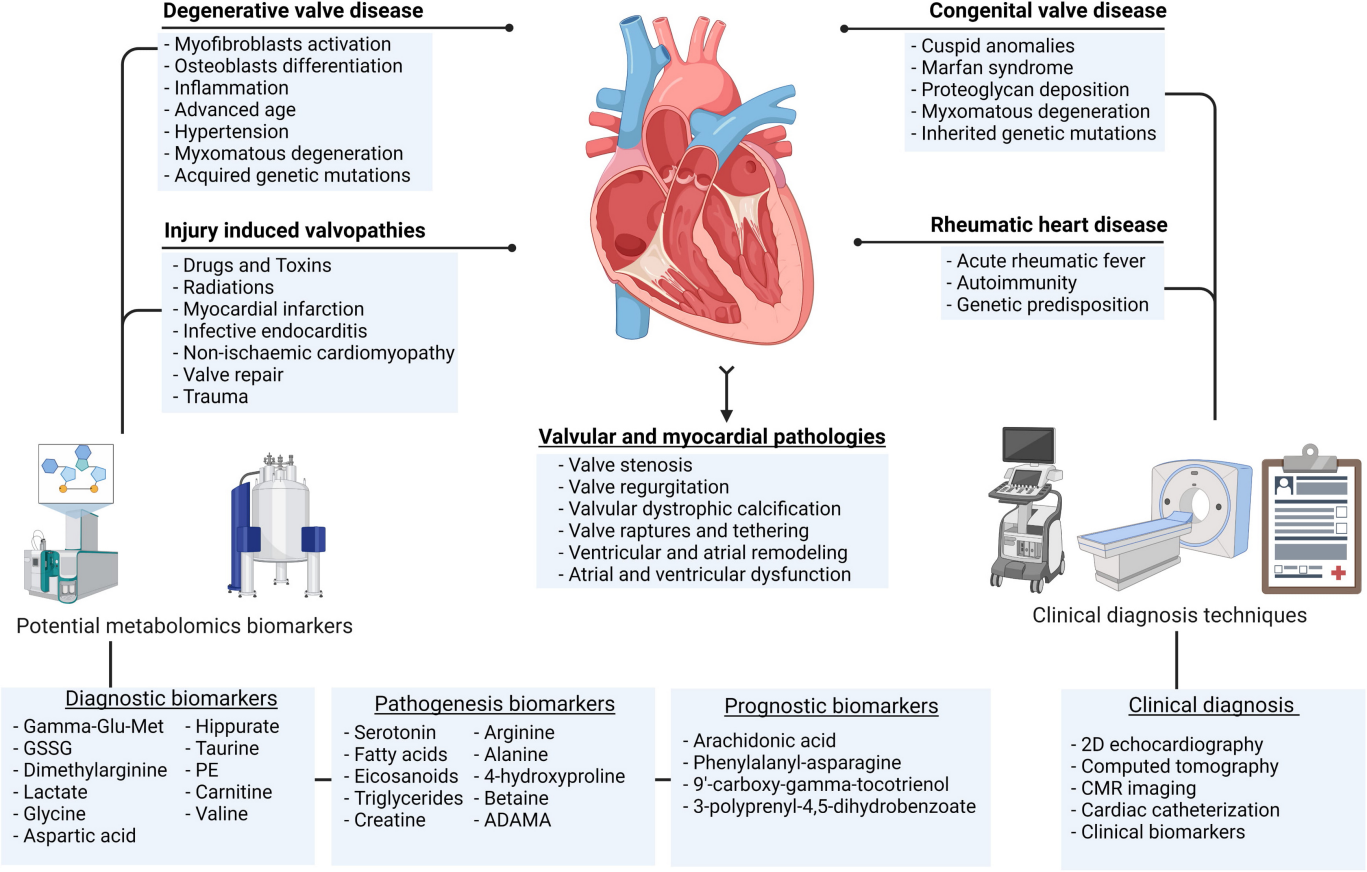
**The common valvular heart diseases, aetiologies, and diagnostic techniques**. Showing the clinical and potential metabolic biomarkers. Gamma-Glu-Met, gamma-glutamylmethionine; GSSG, glutathione disulfide; PE, phosphatidylethanolamine; ADMA, asymmetric dimethylarginine; CMR, cardiac magnetic resonance. Figure created with BioRender.com.

Valvular heart disease(s) (VHD) results from developmental anomalies of cardiac valves and acquired pathology of valvular structure of the heart. VHD are classified into degenerative, congenital, rheumatic, or cardiac injury due to mediastinal radiation exposure, cardiotoxic therapies or carcinoid heart disease. Myocardial infarction, hypertension, age, and hypercholesterolemia are some of the risk factors of acquired VHD (Fig. 1) [9, 10]. The VHD types have varying incidences that depend on the geographic regions and economic status. Central and sub-Saharan Africa (SSA) showed a high age-standardised prevalence of rheumatic heart disease (RHD) (29.40/100,000) as of 2017 and an increase in non-rheumatic VHD from 244.55/100,000 to 247.26/100,000 between 1990 and 2017, respectively [11]. The RHD prevalence trends in Africa and other resource limited regions are considerably higher than to those seen in developed countries such as high-income North America or Western Europe. Africa is also seeing a rise in non-rheumatic VHD and occur against a background of weaker medical infracstructure [11]. The trend in central and sub-Saharan Africa presents a considerable challenge in diagnosis and management of VHD thus simple and reliable biomarkers for early diagnosis are needed.

The only proven treatment for VHD is timely valve replacement or repair, however, it is not always readily available in all geographical regions. There are limited therapeutic strategies available to halt progression of VHD; clinical trials of statins were not successful at halting aortic stenosis (AS) [11]. Metabolomics, therefore, may help in the development of new and more effective targeted therapies. This review highlights the potential of metabolomics in identifying biomarkers which impact on pathogenesis, diagnosis, and prognosis of common VHD.

## 2. Pathogenesis and Clinical Diagnosis of Valvular Heart Disease

**Fig. 2.**
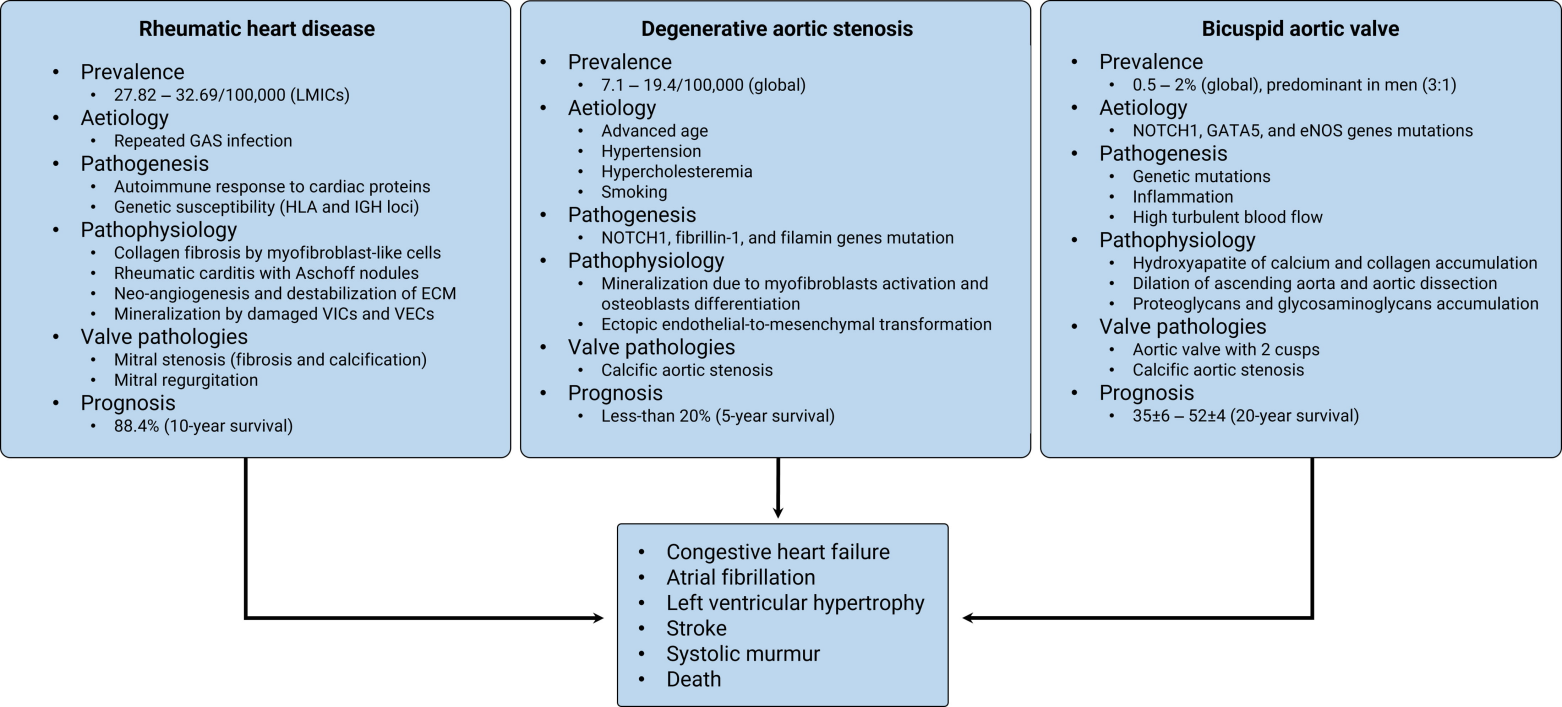
**Summary of RHD, AS and BAV prevalence, aetiologies, pathogenesis, and pathobiology**. LMICs, low- and middle-income countries; GAS, group A Streptococcus; HLA, human leukocyte antigen; IGH, immunoglobulin heavy locus; ECM, extracellular matrix; VICs, valve interstitial cells; VECs, valve endothelial cells; NOTCH1, neurogenic locus notch homolog protein 1; GATA5, GATA binding protein 5; eNOS, endothelial nitric oxide synthase; RHD, rheumatic heart disease; AS, aortic stenosis; BAV, bicuspid aortic valve.

The most common VHD are RHD, degenerative AS, and bicuspid aortic valve (BAV) (Fig. 2). Degenerative AS commonly presents with calcified aortic valves (AV) causing a dilated left atrium (LA) and left ventricle hypertrophy (LVH) [12]. The most common congenital valve lesion is BAV, where the valve has two leaflets, and mostly presents with AS [13]. RHD mostly presents with mitral valve (MV) regurgitation and stenosis, AV regurgitation and stenosis, and tricuspid regurgitation [14]. Mechanical damage of heart valves commonly results in rupture of the valves or chordae tendinae—this can be from biochemical, toxins, radiation, or traumatic injury [15].

Currently, the most accurate method of diagnosing VHD is transthoracic and transoesophageal echocardiography [16, 17]. It is performed at discrete time intervals and is relatively static. On the other hand, dynamic monitoring of cardiac valvular molecular biomarkers may detect ongoing inflammatory and degenerative valvular changes even when echocardiography demonstrates a stable valvular condition.

### 2.1 Rheumatic Heart Disease

RHD is prevalent in SSA (Fig. 2) and is a sequel to acute rheumatic fever (ARF) after pharyngeal or skin infection with group A Streptococcus (GAS) [11, 18]. GAS M proteins induce autoimmune reactions against the host’s cardiovascular proteins [18]. M proteins have similar immunogenic epitopes to hosts’ myosin, actin, tropomyosin, and laminin, which stimulate proinflammatory responses [18]. Genetic susceptibility, low social economic status and overcrowded dwellings are some of the risk factors for ARF and RHD [19]. The immune response leads to the damage of the quiescent fibroblast-like cells which leads to collagen remodeling leading to fibrosis, mineralization, and stiffening of the leaflets [18]. Furthermore, changes in gut and oral microbiota have been associated with RHD severity [20].

### 2.2 Degenerative Aortic Stenosis

Degenerative AS is prevalent in high-income countries (HICs), affecting mainly older persons but the prevalence is rising in low- and middle-income countries (LMICs) [11]. Degenerative AS is characterised by dystrophic calcification, and also associated mitral regurgitation due to myxomatous degeneration [11]. Progression of degenerative AS is linked to activation of myofibroblasts, osteoblast differentiation or high shear forces [21]. Dystrophic aortic calcification is associated with inflammatory activation, advanced age, smoking status, BAV, and hypertension (Fig. 2) [21]. However, advanced age, smoking, and hypertension are independently associated with activation of inflammation pathways [22, 23]. In addition, neurogenic locus notch homolog protein 1 (NOTCH1), fibrillin-1 (FBN1), and filamin (AFLNA) gene mutations are linked with the development of degenerative AS and mitral valve prolapse [21].

### 2.3 Bicuspid Aortic Valve

Common congenital valve defects are the BAV and MV prolapse [11]. BAV predominantly leads to aortic stenosis (AS) and/or regurgitation and calcification [13]. The condition has been shown to be more common in males, with a male-female ratio of about 3:1. BAV results from incomplete separation of the leaflets in the development stage due to defective cushion formation or septation of the outflow tract (Fig. 2) [13]. Pathobiology of BAV is multi-layered including mineralization, inflammation due to disorganised tissue structure, haemodynamic stress, and genetic mutations [13]. The mineralization observed in calcific aortic valve stenosis is linked to cell apoptosis and necrosis that enables dystrophic calcification predominately due to accumulation of hydroxyapatite of calcium [13]. In non-calcified BAV, there are anomalies in the organisation of the valve interstitial cells which lead to accumulation of proteoglycans, glycosaminoglycans and the extra cellular matrix which promotes lipid retention [13]. With regards to the haemodynamic stress, a BAV experiences higher blood flow turbulence as compared to tricuspid aortic valve (TAV) leading to more mechanical stress which has been shown to cause increased collagen deposition and mineralization of the leaflets [13].

## 3. Metabolomics in Valvular Heart Disease

**Fig. 3.**
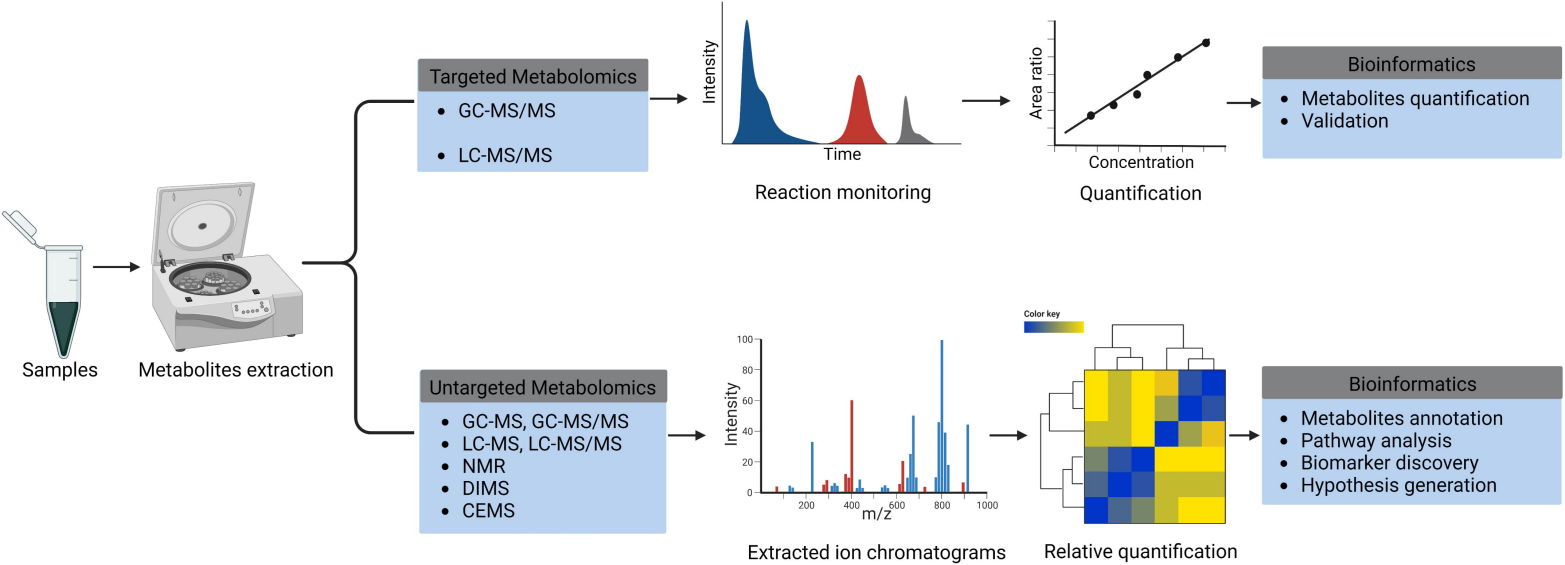
**Schematic summary of targeted and untargeted metabolomics approaches**. GC-MS/MS, gas chromatography with tandem mass spectrometry; LC-MS/MS, liquid chromatography with tandem mass spectrometry; GC-MS, gas chromatography mass spectrometry; LC-MS, liquid chromatography mass spectrometry; NMR, nuclear magnetic resonance; DIMS, direct-infusion mass spectrometry; CEMS, capillary electrophoresis mass spectrometry; *m/z*, mass-to-charge ratio. Figure created with BioRender.com.

Metabolomics is the comprehensive study of small molecules (50–1500 Da) and can measure effects of endogenous and exogenous phenomena which affect phenotype [1]. Considering the proximity to the biologic phenotype, metabolomics holds great potential in objectively measuring and understanding tissue pathophysiological processes, including the impact of multiple genetic, nutritional, and environmental factors. Due to the early pathological changes in metabolic profiles and the technical capabilities to analyse multiple features at once, metabolomics can facilitate in-depth investigations of VHD [24, 25, 26, 27, 28, 29]. Researchers need to decide a priori whether to use targeted or untargeted metabolomics approaches for their studies (Fig. 3).

Targeted experiments are designed for qualitative and quantitative analysis of specific groups of molecules which are either chemically related or belong to the same biological pathway. A targeted approach is suitable for quantification of differences in potential biomarkers between phenotypes [30]. By contrast, untargeted metabolomics measures many metabolites in an unbiased manner, i.e., the chemical extraction and analysis methods are not optimized for specific chemical classes. Untargeted metabolomics is suitable for “hypothesis generating” studies allowing discovery of specific pathways or biomarkers that associate with specific phenotypes [30], assuming such studies are based on a well-designed and testable biological question.

Upon data acquisition (especially in untargeted metabolomics), the raw data is normally processed through automated or semi-automated bioinformatic pipelines [30]. The initial step in the metabolomics data analysis is data pre-processing which converts the graphical spectra into computer useable data formats. Data processing includes normalization, peak detection and quantification, chromatogram alignment (where necessary), and filtering [30]. For untargeted metabolomics, data processing is followed by structural elucidation and annotation/identification, biomarker discovery statistics, and functional analysis.

### 3.1 Applications of Metabolomics in Valvular Heart Disease

**Fig. 4.**
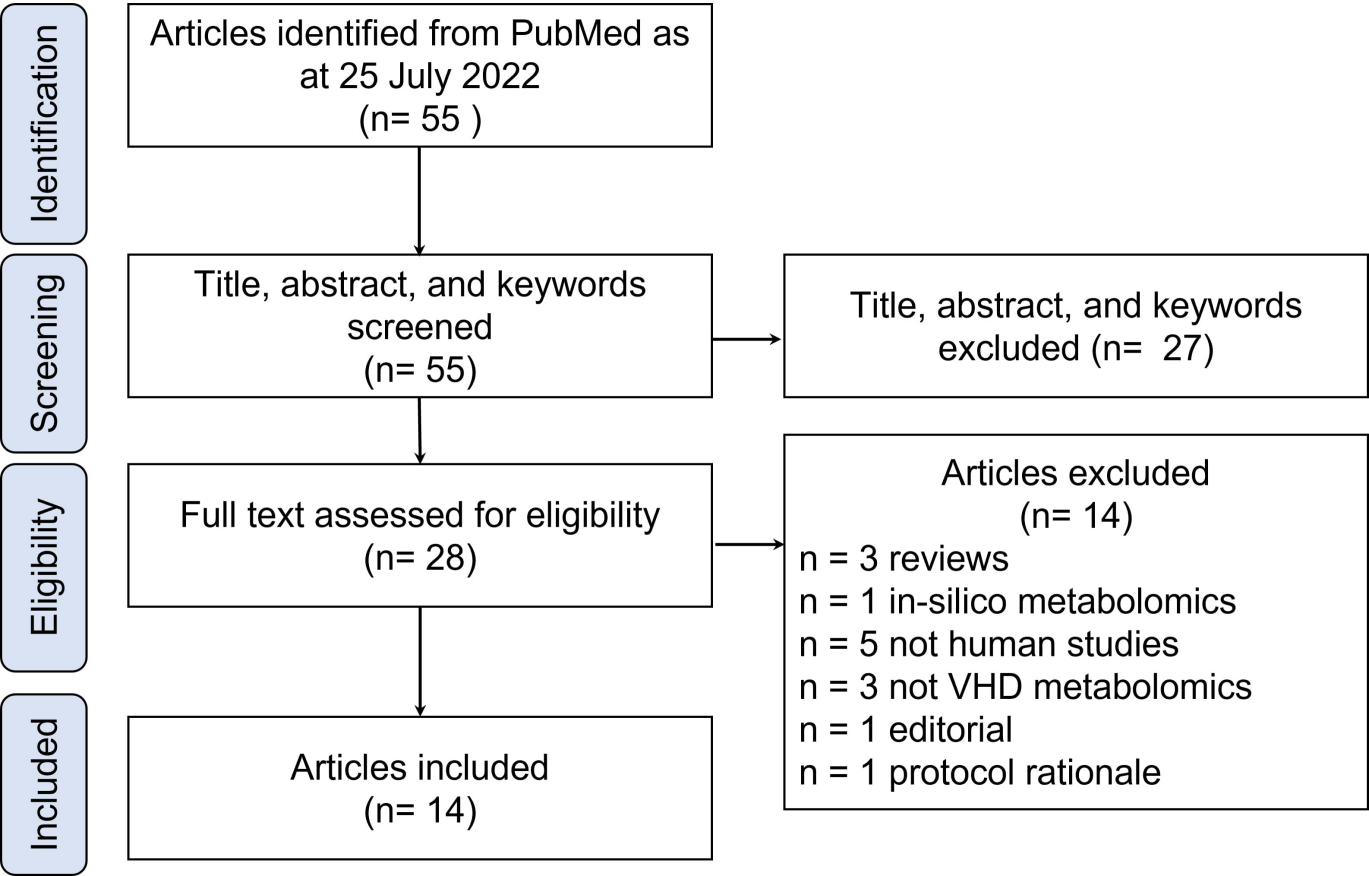
**Flow diagram of systematic review selection criteria**. VHD, valvular heart disease.

We inputted the search terms “metabolomics AND valvular heart diseases OR congenital valve diseases OR bicuspid aortic valve OR degenerative aortic valve OR calcific aortic stenosis OR myxomatous mitral valve disease OR rheumatic heart disease”, into PubMed to identify metabolomics studies of VHD up to 25th July 2022. A total of 55 studies were found, of which 41 were excluded due to being reviews, not being studies on VHD metabolomics, and not being metabolomics studies on human VHD (Fig. 4). The remaining 14 studies were included and reviewed and are summarised in Table 1 (Ref. [24, 25, 26, 27, 28, 29, 31, 32, 33, 34, 35, 36, 37, 38]) and are discussed below.

**Table 1.** **Summary of the reviewed metabolomics studies in valvular heart diseases indicating the sample size, disease phenotypes, approaches and techniques, bio-samples, extraction methods, pathogenesis, diagnostic and prognostic biomarkers, and if the studies are validated**.

Author and year	Sample size	Study Participants	Approach (Techniques)	Bio-sample(s)	Metabolites extraction	Pathogenesis biomarkers	Diagnostic biomarkers	Validated	Reference
Das *et al*. (2022)	100	RHD vs healthy	Untargeted (LC-MS)	Plasma	Methanol (monophasic)	(N-acetylneuraminate, Arachidonic acid, D-Sphingosine, 16(R)-HETE, orotate, inosine, Hypoxanthine, linoleate, Prostaglandin B, d-(+)-Pyroglutamic Acid, l-5-Hydroxytryptophan, Adenosine monophosphate, l-glutamic acid, 5-Methoxysalicylic acid, Prostaglandin A1, d-pantothenic acid, xanthine, (Caprolactam, trans-4-Hydroxy-l-proline, dihydroxymandelic acid, alphaAspartylphenylalanine, 2’-Deoxyuridine, alpha-Lactose, 4-Nitrophenol, 4-Anisic acid	Caprolactam, N-acetylneuraminate, trans-4-Hydroxy-l-proline, Dihydroxymandelic acid	No	[29]
Jiang *et al*. (2019)	154	MVD (MS, MR) vs healthy	Untargeted (NMR)	Plasma	Methanol (monophasic)	Formate, 2-oxoisocaproate, lysine, tryptophan, alanine, lactate, 2-hydroxybutyrate, Octanoate, Acetate, Creatine, Acetone, Calcium, N-Acetyl Glycoproteins	Formate, lactate	No	[26]
Al Hageh *et al*. (2020)	92	AS vs healthy	Untargeted (GC-MS)	Urine and Plasma	Plasma; Methanol, water, and chloroform (biphasic)	-	trans-Aconitic acid, myristic acid, methylmalonic acid, 7-Dehydrocholesterol, 2,4-Di-tert-butylphenol, malonic acid, 2-Hydroxyhippuric acid, 3-Hydroxyhippuric acid, succinic acid, glycerol, quinic acid, uric acid, stearic acid, 4-Deoxyerythronic acid, 3-(3-Hydroxyphenyl)-3-Hydroxypropanoic acid (HPHPA) and myo-inositol	No	[25]
					Urine; methanol (monophasic)				
Elmariah *et al*. (2016)	44	AS with AKI vs AS no AKI	Targeted (LC-MS)	Plasma	acetonitrile/methanol/formic acid (monophasic)	-	5‐adenosylhomocysteine, xanthosine, trimethylamine‐N‐oxide (TMNO), cysteamine, C4‐butyryl carnitine, and C4‐methylmalonyl carnitine, kynurenic acid, xanthosine, TMNO, taurine, asymmetric/symmetric dimethylarginine, cysteamine, short‐chain acyl carnitines, creatinine	No	[38]
Elmariah *et al*. (2018)	44	AS (with & without LVH)	Targeted (LC-MS)	Blood	-	-	acylcarnitines (C16, C18:1, C18:2, C18, C26), choline, kynurenine	No	[37]
Haase *et al*. (2021)	50	High gradient AS vs healthy	Targeted (LC-MS/MS)	Plasma	-	Acylcarnitines, amino acids and biogenic amines, sphingomyelins, PC, LysoPC, and PC	Amino acids and biogenic amines, glycerophospholipids, LysoPCs, PC, SM:PC, LysoPC:PC, acylcarnitines, creatinine, triglycerides, alanine	No	[36]
Mourino-Alvarez *et al*. (2016)	44	AS vs AR	Untargeted and targeted, Multi-omics (GC-MS)	Plasma	ACN (monophasic)	serine, citric acid, tartronic acid, 6-octadecanoate-a-D-glucopyranoside, succinic acid, 5-hydroxytryptophan, isoleucine, malic acid, aspartic acid, aminomalonic acid, leucine, gluconic acid, alanine, threonine, 1-monolinolein, pyroglutamic acid, tetrahydroxypentanoic acid 1,4-lactone,glycine and sorbitol pyroglutamic acid and succinic acid, alanine	Pyroglutamic acid, succinic acid, alanine	Yes	[31]
Olkowicz *et al*. (2017)	85	Degenerative AS vs healthy	Targeted, Multi-omics (IP-RPLCMS/MS, Shortgun LC-MS/MS proteomics	Plasma	ACN (monophasic)	Arginine, Homo-L-arginine, Asymmetric dimethylarginine, Symmetric dimethylarginine, 4-Hydroxyproline, Betaine, 3-Methylhistidine	-	No	[33]
Surendran *et al*. (2020)	106	CAS stages	Targeted and untargeted (LC-MS and LC-MS/MS)	Aortic valve biopsies	Methanol, acetonitrile, and water (monophasic) chloroform and methanol (biphasic)	Triglycerides, random glucose, creatine, LysoPE, MG, LysoPA, pyridinoline, glycoursodeoxycholic acid, LysoPC, PC	LysoPAs	No	[32]
van Driel *et al*. (2021)	19	AS vs healthy	Untargeted (DI-HRMS)	Serum	Methanol (monophasic)	9’-carboxy-gamma-tocotrienol, 3-polyprenyl-4,5-dihydroxybenzoate, asparaginyl-Phenylalanine	(phenylalanyl-asparagine, dihydropteridine, alpha-tocotrienol, 9’-carboxy-gamma-tocotrienol, 3-hydroxymelatonin, 3-polyprenyl-4,5-dihydroxybenzoate, Prostaglandin F1a, alpha-linolenyl carnitine, 14-HDoHE, 24,25,26,27-Tetranor-23-oxohydroxyvitamin D3, 11beta,20-Dihydroxy-3-oxopregn-4-en-21-oic ${\mathrm{acid)}}^{1}$	No	[24]
Xiong *et al*. (2020)	57	BAV AS vs TAV AS	Untargeted (GC and LC-MS)	Plasma	Methanol (monophasic)	L-Glutamine, L-Proline, Hydroxyproline, pyrrile-2-carboxylic acid, NS-Succinyl-L-ornithine, spermine, (L-Glutamine, L-Arginine, Pyruvic acid, Homocarnosine, ${\mathrm{Ornithine)}}^{2}$	(6-Keto-prostaglandin F1a, Leukotriene B4, Arachidonic acid, Leukotriene ${\mathrm{E4)}}^{3}$, (15-KETE, 15(S)-HETE, arachidonic acid, prostaglandin G2, Thromboxane B2, Leukotriene A4, Leukotriene ${\mathrm{B4)}}^{4}$	No	[28]
Martinez-Micaelo *et al*. (2020)	212	BAV vs TAV	Untargeted (LC-MS)	Plasma	Methanol and dichloromethane (biphasic)	Alpha-Tocopherol, choline	Alpha-tocopherol	No	[27]
Chessa *et al*. (2021)	44	BAV vs healthy	Untargeted (NMR)	Urine	-	3-hydroxybutyrate, Alanine, Creatine, Glycine, Hippurate, Taurine, Betaine	Glycine, Hippurate, Taurine	No	[34]
Wang *et al*. (2016)	100	BAV vs healthy	Untargeted (LC-MS)	Serum	Methanol (monophasic)	-	Glycerophospho-N-oleyl ethanolamine, monoglyceride, phosphatidylethanolamine	No	[35]

^1^Prognostic biomarkers for ventricular reverse remodelling post-AVR, ^2^Pathogenesis biomarkers that reversed expression post-TAVR, ^3^Prognostic biomarkers changed pre-TAVR, ^4^Prognostic biomarkers changed post-TAVR. ACN, acetonitrile; AS, aortic valve stenosis; CAS, calcific aortic stenosis; AKI, acute kidney injury; LC-MS, liquid chromatography mass spectrometry; LC-MS/MS, liquid chromatography with tandem mass spectrometry; GC-MS, gas chromatography mass spectrometry; NMR, nuclear magnetic resonance; IP-RPLCMS/MS, ion-pairing reversed-phase liquid chromatography with tandem mass spectrometry; DI-HRMS, direct-infusion high-resolution mass spectrometry; LVH, left ventricle hypertrophy; BAV, bicuspid aortic valve; TAV, tricuspid aortic valve; MVD, mitral valve disease; MS, mitral valve stenosis; MR, mitral valve regurgitation; KETE; keto-eicosatetraenoic acid; HETE, hydroxyeicosatetraenoic acid; PC, phosphatidylcholine; RHD, rheumatic heart disease; SM, sphingomyelines.

#### 3.1.1 Pathogenetic Biomarkers

Applying metabolomics methods to VHD pathologies is underutilised. In the preceding ten years up to 2020, between zero and eight papers a year were referenced in PubMed on this subject. However, in 2021, at least 19 papers were published, and there is a slow overall upward trend. Major focuses of investigations include circulatory and tissue-specific biomarkers together with their related pathways and genes.

To the best of our knowledge, few studies have explored the metabolic profiles in RHD. However recently, Das and colleagues have reported dysregulation of metabolites involved in Purine, Glutamine, Glutamate, Pyrimidine, Arginine, Proline and Linoleic metabolic pathways in rheumatic heart disease patients [29]. Like other severe valvular heart diseases, the dysregulated pathways were mostly energetic and amino acid metabolism pathways. Further, the involvement of linoleic acid metabolism may suggest proinflammatory processes in RHD since it has previously been linked to activation of vascular endothelial cells [29]. As indicated earlier, RHD most often affects the mitral valve leading to mitral valve stenosis or regurgitation. Mitral valve disease has been associated with dysregulation of inflammatory processes, energy metabolism, amino acid, and calcium metabolism. Further, serotonin and branched chain amino acids were reported to be dysregulated in both humans and canines [26]. The dysregulation of serotonin and related amino acids may suggest involvement of autocrine serotonin signaling in myxomatous mitral valves [26]. Further, the dysregulation of the autocrine system and fatty acids in valvular heart conditions may explain the increased rates of depression among heart disease patients [39].

Mourino-Alvarez used metabolomics to study AS. Metabolites involved in the alanine pathway and immune response processes were reported to be dysregulated in patients with AS [31]. Similar findings were reported by a multi-omics study that found dysregulation of inflammation proteins, lipids dysregulation, and changed amino acid profiles in AS patients [33]. Inflammation is thought to have a significant contribution towards worsening of calcification as is also seen in atherosclerosis [40]. Metabolic signatures have also shown a strong correlation with clinical parameters for valve morphologies, VHD severities, and classical markers of cardiac injury [24, 28, 33]. Surendran *et al*. [32] investigated the tissue-specific metabolic profiles in patients at different stages of calcific aortic valve stenosis (CAS), i.e., mild to severe CAS. Their findings suggested that pathways involved in lipid metabolism and biosynthesis are mostly associated with CAS severity [32]. Specifically, LysoPE, monoacylglyceride (MG), and LysoPA and their metabolic species showed the strongest associations with CAS severity [32]. From their findings, LysoPA was strongly implicated as a factor in the rate of CAS progression [37]. In a similar study, dysregulation of nitric oxide synthesis, fatty acids, and tetrahydrobiopterin metabolism was reported post-aortic valve replacement (AVR) in CAS [24]. Dysregulation of fatty acids and eicosanoids may be indicative of inflammatory processes in patients with severe AS in a similar process to atherosclerosis [24]. Interestingly, the levels of antioxidant metabolites, NO metabolism metabolites, and steroids involved in inflammatory pathways reversed toward healthy control levels 4 months post-AVR [24]. Such reversals post-valve replacement may either suggest that they are involved in the worsening of the valve pathologies, or they may represent adaptive strategies to protect the heart or body from the consequences of cardiac insufficiency.

With regards to bicuspid aortic valve disease (BAV), dysregulation of urinary metabolites which map to glycine, serine and threonine metabolism, and the taurine metabolic pathway were associated with its pathologies [34]. In addition, Martinez-Micaelo and colleagues [27] reported involvement of alpha-tocopherol and choline pathways while comparing stenotic bicuspid and tricuspid aortic valves with and without dilatation. The dysregulated pathways suggest a role for inflammation, oxidative stress, and endothelial damage in congenital aortic valve pathologies [27]. In addition, Xiong and colleagues [28] reported valve-specific differences in dysregulation of metabolic biomarkers mapping to arginine and proline metabolic pathways both before-transthoracic aortic valve replacement (TAVR) and 7 days post-TAVR in BAV and tricuspid aortic valves, and that arachidonic acid may be predictive of poorer haemodynamics following surgery in BAV.

#### 3.1.2 Diagnostic Biomarkers

Diagnostic tools and guidelines already exist to identify VHD [16, 17]. However, early detection and screening are challenging. Several metabolomics studies have investigated metabolic biomarkers that could be used for early detection and diagnosis of VHD. Caprolactam, N-Acetylneuraminate, arachidonic acid, L-5-Hydroxytryptophan, D-Pantothenic acid, and 4-Nitrophenol showed good performance in distinguishing RHD patients from healthy individuals [29]. Additionally, Jiang and colleagues [26] reported formate and lactate as having very good performance as diagnostic biomarkers for mitral stenosis and mitral regurgitation with high sensitivity, and specificity.

Further, a study that compared plasma and urine metabolic profiles reported biomarkers with capabilities of differentiating aortic valve stenosis patients from healthy controls [25]. It was also one of the few studies that investigated the comparability of different biofluids in their utility as biomaterials for biomarker research [25]. Their findings showed that biomarkers detected in plasma were in agreement to those detected in urine and had excellent biomarker performance after metabolomics data were normalized to creatinine levels [25].

Urinary glycine, hippurate, and taurine showed good diagnostic performance at differentiating patients with BAV from healthy individuals [34]. Urine has the distinct advantage that it can be collected at home and is pain free to collect. In addition, based on logistic regression and receiver operating characteristic (ROC) curve analysis results, Wang and colleagues [35] reported serum glycerophospho-N-oleyl ethanolamine, monoglyceride, and phosphatidylethanolamine as suitable biomarkers to diagnose BAV patients from healthy participants. Their findings indicate that dysregulation in lipids and lipoprotein metabolism are the main drivers of endothelial damage and inflammation in calcific BAV. In addition, a proteomics and metabolomics study reported a panel of proteins and metabolites associated with “coagulation, inflammation and immune response”, “response to ischaemia”, and lipid metabolism as potential discriminatory biomarkers between calcific aortic stenosis and aortic regurgitation [31]. Metabolic profiling could also be used for sensitive screening procedures by associating it with specific valvular morphologies. A recent study used random forest prediction to show that alpha-tocopherol is a potential metabolic biomarker capable of predicting aortic valve morphology or dilation of the ascending aorta in BAV patients [27]. Combining alpha-tocopherol, endothelial microparticles (EMPs) and C-reactive proteins (CRP) showed a strong ROC specificity and allowed for the discrimination of aortic morphologies of the studied patients [27].

#### 3.1.3 Prognostic Biomarkers

Since it is often challenging to predict patients’ outcomes post valve replacement intervention using conventional means, there is a need for biomarkers with high prognostic accuracy. However, there are very few studies that have investigated multivariate metabolic biomarkers for predicting outcomes post valve repair or replacement. A targeted metabolomics study following surgery observed a decrease in formerly elevated amino acids, biogenic amines, and glycerophospholipids to levels approaching clinically healthy patients, suggesting their involvement in worsening of aortic valve pathology in patients with high gradient aortic stenosis [36]. Specifically, metabolites belonging to the glycerophospholipids class were reversed post-TAVR to healthy control levels; glycerophospholipid metabolism perturbation is associated with dysregulation of inflammatory processes [36]. Further, while correlating the dysregulated biomarkers to the clinical parameters pre- and post-TAVR showed a strong association between acylcarnitine, alanine and phosphatidylcholines (PCs) with changes in left ventricular ejection fraction (LVEF), left ventricular end-diastolic diameter (LVEDD), left ventricular mass index (LVMI), and left ventricular posterior wall thickness in diastole (LVPWD) suggesting that the metabolites could predict reverse remodelling post-TAVR [36]. Delayed valve replacement in patients with severe AS may lead to irreversible left ventricle (LV) remodelling. Elmariah *et al*. [37] showed long chain acylcarnitines as suitable predictors of LV reverse remodeling after AVR. Long chain acylcarnitines (C16, C18:1, C18:2, and C18) were decreased in AS patients 24 hours post-AVR [37]. In another study, Elmariah and colleagues used plasma metabolic profiles to predict AS patients’ likelihood of dying from acute kidney injury (AKI) post-TAVR [38]. Elevated S-adenosylhomocysteine was associated with development of AKI and predicted mortality up to 7.8 months post-TAVR [38]. Xiong and colleagues [28] observed that a combination of poor hemodynamics and reduced ventricular function before-TAVR combined with dysregulation of arachidonic acid metabolism pathways post-TAVR was associated with worse outcome and reduced reverse remodelling. This may suggest that arachidonic acid metabolism could be playing a critical role in the worsening of ventricular function and delayed ventricular reverse remodelling post-intervention. Xiong *et al*. [28] showed that therapeutically targeting arachidonic acid metabolism protected against heart failure, decreased myocardial fibrosis, and led to regained myocardial function. In summary, the reported prognostic biomarkers indicate that metabolomics has potential in providing biomarkers that may dictate treatment options.

### 3.2 Potential Pitfalls and Limitations of Metabolomics in VHD

The success of metabolomics experiments rides on the experimental design and sample collection. Metabolomics is a multi-disciplinary study and bringing disciplines together to plan studies at the earliest possible stage is important. Careful study design and sample collection avoid false discoveries due to poor sample handling and storage, or confounding factors overinfluencing results [41]. Good study design starts with a well framed hypothesis, or biological question which is both defined and testable.

Analytically, some of the common pitfalls in metabolomics studies can occur during sample preparation, analysis, statistics, and reporting of biomarkers. For chemical analysis using certain techniques such as mass spectrometry, ion suppression or enhancement is a common phenomenon and often seen in complex matrices such as plasma or tissues, and it can mask detection of metabolites of interest; effects can be reduced by using internal standards, serial dilution or prior matrix clean up and separation technologies [42]. However, matrix effect differences can be particularly pronounced in studies where the physiology between the two classes may be very different (e.g., particularly high haematocrit levels in heart failure, or high glucose in diabetics).

Discovery of spurious biomarkers is highly likely due to statistical errors of chance, especially if analysing many hundreds of variables concurrently [30]. This phenomenon is seen wherever multiple testing is done and occurs because most medical studies typically allow a 5% chance of a false positive result (*p* = 0.05 as a cut off). This becomes additive the more tests are undertaken, such that, by the time you have conducted 100 tests, on average, five of them will be positive purely by chance [30]. Validation of statistical models is important, and false discovery correction is commonly employed in univariate methods to adjust p-values based on how many tests were undertaken. This has the unfortunate effect of potentially increasing the type II error rate, i.e., increasing the number of false negatives. This highlights the importance of appropriately powered sample sizes, independent cohorts, and targeted analyses to follow up important results.

Full identification of biomarkers in untargeted metabolomics remains a great challenge and normally requires nuclear magnetic resonance (NMR), but it could be mitigated by validating the putatively annotated biomarkers using orthogonal factors such as exact mass, mass spectral fragmentation data, retention time of the unknown features, prior knowledge of the kind of metabolites expected in the sample, and the isotopic peak envelopes of the annotated features [42]. In the last ten years, the advent of chemical-rule based algorithms and machine learning to predict de novo structures, along with the availability of well curated mass spectral fragment libraries and annotation matching software have greatly improved the annotation rate.

Many studies reporting metabolic biomarkers involved in pathogenesis often find it challenging to ascribe causality of circulatory biomarkers to cardiac pathologies [43, 44]. Some of the circulatory biomarkers could be indicators or epiphenomena of metabolic disturbances in other organs other than the heart, or of altered gut microbiomes that contribute towards immune and cardiac pathologies. Some of the studies reviewed here describe metabolites associated with specific valvular pathologies where the included patients had heart failure [28, 31]. It is a well-known phenomenon that patients with severe VHD may present with heart failure [12, 13, 14, 15]. However, the affect heart failure may have on the observed metabolite changes remains to be investigated. Therefore, follow-up validation experiments with knockout models are encouraged where affected pathways could be disrupted to ascribe causality to the observed pathologies. Studies with small sample sizes are another limitation. The cost of collecting and curating large sample sizes, and the complexities of accessing invasive tissues remain a challenge [31, 45].

Even fewer studies investigate disease phenotypes at different stages of development such as mild and severe VHD, or at best, conduct longitudinal studies to trace metabolic shifts over a time course [46]. Longitudinal studies and those including different disease stages would provide significant insight into the magnitude and direction of dysregulation of the potential biomarkers but are often further confounded with small sample sizes in each disease stage. To the best of our knowledge, studies comparing circulatory and tissue-specific metabolic profiles in VHD to determine the reliability of using circulatory biomarkers to understand cardiovascular diseases are sparse.

## 4. Conclusions

Congenital, degenerative, rheumatic, or mechanical valvopathies have different aetiologies and pathogenesis, but mostly lead to similar pathophysiology which remains challenging to detect in the early stages. Metabolomic techniques have been used for discovery and quantification of diagnostic biomarkers and to identify those that have an impact on cardiovascular diseases pathogenesis. The field of metabolomics has seen a steady improvement in analytical technologies and development of tools for data processing and analysis. We have summarised studies that report on metabolic biomarkers which describe the pathogenesis of calcific aortic stenosis, degenerative mitral valve stenosis, rheumatic heart disease, and congenital valvular heart diseases. We have summarised biomarkers used for diagnosis and prediction of post-intervention outcomes in BAV, CAS, and mitral valve diseases. We have also highlighted potential limitations and pitfalls that are common in metabolomics studies. To the best of our knowledge there are very few metabolomics studies that have investigated rheumatic valve diseases despite it being endemic in developing countries. The study of metabolomics could be of interest to improve understanding of the pathogenesis and prognosis of VHD that are endemic in LMICs. However, it will be of great importance to assess how much metabolomics changes is relate to a specific VHD or are rather a reflection of the associated heart failure. Studies on VHD with specific degrees of heart failure as well as on heart failure not due to valvular heart disease may help to assess if metabolomics may be proposed as a diagnostic and prognostic biomarker in the specific field of VHD.
